# Correction: Combining organic and mineral fertilizers as a climate-smart integrated soil fertility management practice in sub-Saharan Africa: A meta-analysis

**DOI:** 10.1371/journal.pone.0295990

**Published:** 2023-12-12

**Authors:** Gil Gram, Dries Roobroeck, Pieter Pypers, Johan Six, Roel Merckx, Pauline Chivenge, Bernard Vanlauwe

Pauline Chivenge should be included in the author byline instead of the Acknowledgments. Pauline Chivenge should be listed as the sixth author, and their affiliation is 5: International Rice Research Institute (IRRI), Manila, Philippines.

The correct citation is: Gram G, Roobroeck D, Pypers P, Six J, Merckx R, Chivenge P, et al. (2020) Combining organic and mineral fertilizers as a climate-smart integrated soil fertility management practice in sub-Saharan Africa: A meta-analysis. PloS ONE 15(9): e0239552. https://doi.org/10.1371/journal.pone.0239552
