# Supplementary material for: Combining organic and mineral fertilizers as a climate-smart integrated soil fertility management practice in sub-Saharan Africa: A meta-analysis
Source: PLoS One. 2020 Sep 24;15(9):e0239552. doi: 10.1371/journal.pone.0239552 (PMC7514003; doi:10.1371/journal.pone.0239552)
Supplement: S1 Table — Run by the rma.mv function of the Metafor package in R [117]. (PDF) [file pone.0239552.s001.pdf]

S1 Table. Output from the YIELD model. Run by the *rma.mv* function of the Metafor package in R [117]

	Multivariate Meta-Analysis Model (k = 2943; method: REML)							
	logLik	Deviance	AIC	BIC	AICc			
	-342.3623	684.7246	752.7246	956.0338	753.5493			
	Variance Components:							
		estim	sqrt	nlvls	fixed	factor		
	sigma^2.1	0.0924	0.304	40	no	ref		
	sigma^2.2	0.0251	0.1583	2943	no	idRow		
	outer factor:	idSite	(nlvls = 71)					
	inner factor:	treatment	(nlvls = 4)					
		estim	sqrt	nlvls	fixed	factor		
	tau^2.1	0.1865	0.4319	275	no	Control		
	tau^2.2	0.0922	0.3036	374	no	MR		
	tau^2.3	0.1035	0.3217	1039	no	OR		
	tau^2.4	0.064	0.253	1255	no	ORMR		
	rho	0.8154			no			
	outer factor:	idSite.time	(nlvls = 271)					
	inner factor:	treatment	(nlvls = 4)					
		estim	sqrt	nlvls	fixed	factor		
	gamma^2.1	0.1072	0.3274	275	no	Control		
	gamma^2.2	0.1429	0.378	374	no	MR		
	gamma^2.3	0.1129	0.336	1039	no	OR		
	gamma^2.4	0.1405	0.3748	1255	no	ORMR		
	phi	0.9435			no			
	Test for Residual Heterogeneity: QE(df = 2921) = 1068664.4274, p-val <.0001							
	Test of Moderators (coefficients 2:22): QM(df = 21) = 1259.6581, p-val <.0001							
	Model Results:							
		estimate	se	zval	pval	ci.lb	ci.ub	
1	intrcpt	1.43739062	0.07320632	19.6347876	7.80E-86	1.29390886	1.58087237	***
2	rateORone	0.00444954	0.0004299	10.3502269	4.17E-25	0.00360695	0.00529212	***
3	rateORtwo	0.00423038	0.00035539	11.9035562	1.13E-32	0.00353383	0.00492693	***
4	rateORthree	0.00358123	0.0009227	3.88126081	0.00010392	0.00177277	0.00538968	***
5	rateORManure	0.00343231	0.00031124	11.0278798	2.80E-28	0.00282229	0.00404233	***
6	kgMN	0.00808516	0.00065473	12.3488246	4.94E-35	0.00680191	0.0093684	***
7	I(rateORone^2)	-6.28E-06	1.29E-06	-4.8539247	1.21E-06	-8.82E-06	-3.74E-06	***
8	I(rateORtwo^2)	-7.38E-06	9.45E-07	-7.8145276	5.52E-15	-9.23E-06	-5.53E-06	***
9	I(rateORthree^2)	-2.11E-05	6.39E-06	-3.30309	0.00095626	-3.36E-05	-8.58E-06	**
10	I(rateORManure^2)	-3.96E-06	6.24E-07	-6.3437568	2.24E-10	-5.19E-06	-2.74E-06	***
11	I(kgMN^2)	-2.95E-05	4.01E-06	-7.3651538	1.77E-13	-3.74E-05	-2.17E-05	***
12	cropSysintercrop	-0.2805024	0.03668726	-7.6457718	2.08E-14	-0.3524081	-0.2085967	***
13	cropSysrotation	0.64659113	0.57716484	1.12028849	0.26259084	-0.4846312	1.77781343	
14	idFNPK	-0.0069753	0.05154152	-0.1353332	0.89234842	-0.1079948	0.09404424	
15	rateORone:kgMN	-1.83E-05	5.50E-06	-3.3203671	0.00089899	-2.90E-05	-7.48E-06	***
16	rateORtwo:kgMN	-1.86E-05	3.99E-06	-4.657516	3.20E-06	-2.64E-05	-1.08E-05	***
17	rateORthree:kgMN	-2.52E-05	1.13E-05	-2.233079	0.02554374	-4.73E-05	-3.08E-06	*
18	rateORManure:kgMN	-1.22E-05	3.86E-06	-3.1699561	0.00152462	-1.98E-05	-4.67E-06	**
19	I(rateORone^2):I(kgMN^2)	2.23E-10	1.39E-10	1.60436759	0.10863305	-4.95E-11	4.96E-10	

20	I(rateORtwo^2):I(kgMN^2)	2.34E-10	8.53E-11	2.73977537	0.00614812	6.65E-11	4.01E-10	**
21	I(rateORthree^2):I(kgMN^2)	7.80E-10	6.63E-10	1.1774496	0.2390161	-5.19E-10	2.08E-09	
22	I(rateORManure^2):I(kgMN^2)	1.03E-10	6.43E-11	1.60928133	0.10755484	-2.25E-11	2.29E-10	
	Signif. codes: 0 (***) 0.001 (**) 0.01 (*) 0.05 (.) 0.1 ( ) 1							
