# Supplementary material for: Combining organic and mineral fertilizers as a climate-smart integrated soil fertility management practice in sub-Saharan Africa: A meta-analysis
Source: PLoS One. 2020 Sep 24;15(9):e0239552. doi: 10.1371/journal.pone.0239552 (PMC7514003; doi:10.1371/journal.pone.0239552)
Supplement: S2 Table — Run by the lmer function of the lme4 package in R [99]. Modeled estimates are presented for dSOC, along with p-values for significance [118] and 95% confidence intervals. Extracted and formatted with the stargazer package [119]. (PDF) [file pone.0239552.s002.pdf]

S2 Table. Output from the N-SOC model. Run by the *lmer* function of the lme4 package in R [99]. Modeled estimates are presented for dSOC, along with p-values for significance [118] and 95% confidence intervals. Extracted and formatted with the stargazer package [119]

	Dependent variable:
	dSOC
cumtONone	0.1304^{***} (0.1003, 0.1606)
cumtONtwo	0.1246^{***} (0.0890, 0.1602)
cumtONthree	0.3011^{***} (0.1331, 0.4691)
cumtONfour	0.8082^{*} (0.1623, 1.4541)
cumtONManure	0.1016^{***} (0.0765, 0.1267)
cumtMN	0.0466 (−0.0090, 0.1022)
SOCi	-0.3569^{***} (−0.5031, −0.2107)
I(cumtONoneˆ2)	-0.0103^{***} (−0.0140, −0.0065)
I(cumtONtwoˆ2)	-0.0108^{***} (−0.0149, −0.0066)
I(cumtONthreeˆ2)	-0.0948^{*} (−0.1779, −0.0117)
I(cumtONfourˆ2)	−0.9649 (−2.1169, 0.1872)
I(cumtONManureˆ2)	-0.0048^{***} (−0.0066, −0.0029)
I(cumtMNˆ2)	−0.0165 (−0.0347, 0.0016)
idFNPK	0.0210 (−0.0688, 0.1108)
cumtONone:cumtMN	0.0022 (−0.0048, 0.0091)
cumtONtwo:cumtMN	0.0034 (−0.0037, 0.0105)
cumtONthree:cumtMN	0.0110 (−0.0196, 0.0416)
cumtONfour:cumtMN	0.0159 (−0.0962, 0.1279)
cumtONManure:cumtMN	0.0012 (−0.0034, 0.0058)
Constant	0.3351^{*} (0.0260, 0.6443)
Observations	531
Log Likelihood	76.5937
Akaike Inf. Crit.	−107.1875
Bayesian Inf. Crit.	−8.8679
Note:	*p<0.05; **p<0.01; ***p<0.001
