# Supplementary material for: Combining organic and mineral fertilizers as a climate-smart integrated soil fertility management practice in sub-Saharan Africa: A meta-analysis
Source: PLoS One. 2020 Sep 24;15(9):e0239552. doi: 10.1371/journal.pone.0239552 (PMC7514003; doi:10.1371/journal.pone.0239552)
Supplement: S3 Table — Run by the lmer function of the lme4 package in R [99]. Modeled estimates are presented for dSOC, along with p-values for significance [118] and 95% confidence intervals. Extracted and formatted with the stargazer package [119]. (PDF) [file pone.0239552.s003.pdf]

S3 Table. Output from the C-SOC model. Run by the *lmer* function of the lme4 package in R [99]. Modeled estimates are presented for dSOC, along with p-values for significance [118] and 95% confidence intervals. Extracted and formatted with the stargazer package [119]

	Dependent variable:
	dSOC
cumTocORone	0.0042^{***} (0.0030, 0.0054)
cumTocORtwo	0.0033^{***} (0.0020, 0.0045)
cumTocORthree	0.0019^{**} (0.0006, 0.0033)
cumTocORfour	0.0012 (−0.0002, 0.0025)
cumTocORManure	0.0048^{***} (0.0034, 0.0061)
cumtMN	0.0224 (−0.0051, 0.0499)
SOCi	-0.3658^{***} (−0.5191, −0.2125)
idFNPK	0.0294 (−0.0671, 0.1260)
cumTocORone:cumtMN	−0.0004 (−0.0010, 0.0001)
cumTocORtwo:cumtMN	−0.0003 (−0.0008, 0.0003)
cumTocORthree:cumtMN	−0.0002 (−0.0007, 0.0004)
cumTocORfour:cumtMN	−0.0003 (−0.0008, 0.0003)
cumTocORManure:cumtMN	−0.0003 (−0.0008, 0.0003)
Constant	0.3464^{*} (0.0179, 0.6749)
Observations	488
Log Likelihood	18.8483
Akaike Inf. Crit.	−3.6965
Bayesian Inf. Crit.	67.5388
Note:	$^{*}p<0.05$; $^{**}p<0.01$; $^{***}p<0.001$
