# Supplementary material for: Combining organic and mineral fertilizers as a climate-smart integrated soil fertility management practice in sub-Saharan Africa: A meta-analysis
Source: PLoS One. 2020 Sep 24;15(9):e0239552. doi: 10.1371/journal.pone.0239552 (PMC7514003; doi:10.1371/journal.pone.0239552)
Supplement: Attachment — Submitted filename: Response to Reviewers.docx [file pone.0239552.s005.docx]

Response to reviewers

Rebuttal letter

Dear Editor, dear reviewers,

Thank you for your consideration and review of our manuscript “Combining organic and mineral fertilizers as a climate-smart integrated soil fertility management practice in sub-Saharan Africa: a meta-analysis”. Below we address the review comments, as stated in the submission response email of July 16^th^, 2020 (lines colored in purple refer to the revised document with track-changes):

Reviewer #1: The manuscript entitled “Combining organic and mineral fertilizers as a climate-smart integrated soil fertility management practice in sub-Saharan Africa: a meta-analysis” presents interesting analysis of various nutrients application for Maize production.
Following are major concerns:

**1. What does ‘SE’ represent in the abstract? Put the abbreviation at the correct place in the sentence.**

We do not find ‘SE’ anywhere in the abstract, nor in the rest of the manuscript. Perhaps ‘AE’ was meant? The latter abbreviation was firstly introduced in the abstract, correctly as “agronomic efficiency of N (AE)”, but was indeed not explained at first instance in the introduction. This has been corrected (lines 32-33 and 37).

**2. The authors need to improve the brevity of the manuscript.**

Along with the editing and rephrasing as a response to the other comments and questions, we:

- Removed a ‘,’ from ‘Maize is the primary staple food of more than 300 million people in SSA and an important source of income for particularly smallholder farmers \cite{Shiferaw2011}.’ (line 19)
- Made impact plural: ‘Low productivity and climate change impacts require climate-smart agriculture (CSA), […]’ (line 27)
- Rephrased: ‘an increased soil organic matter content also improves other soil functions such as soil biological processes and soil moisture regime \cite{SangingaWoomer2009}. This, in turn, improves resilience to droughts.’ (line 44)
- Rephrased: ‘Similarly, high N input rates could exceed crop demand and lead to negative interactions, suggesting that N input rates may also have an effect on interactive effects \cite{Gentile2010, Chivenge2011}. ’ (lines 55-57)
- Rephrased: ‘Raising SOC stocks seems, however, to be challenging. Long-term organic and mineral nutrient trials on maize monocropping in SSA consistently reported declining SOC \cite{Diels2004, Mucheru-Muna2014, Kapkiyai1999, Kibunja2012, Sommer2018}, but found that treatments with organic inputs were more successful in reducing SOC losses than mineral and no-input treatments \cite{Akinnifesi2007, Ayuke2011, Bedada2014, Kapkiyai1999, Kibunja2012}.’ (lines 77-79)
- Corrected for American English: ‘Yield data were modeled […]’ (line 170)
- Removed ‘estimated’ (line 301)
- Removed ‘estimated’ (line 339)
- Rephrased: ‘Our analyses showed that ORMR treatments lead to positive interactions, and to AEs and yields that can outperform those of MR treatments.’ (line 374)
- Rephrased: ‘Like other studies, they revealed potentially greater yield responses for the combined application treatments \cite{Gentile2008, Gentile2009, Mafongoya2006, Nziguheba2002}, but could not find evidence for improved AE as predominantly negative interactive effects were observed between the two resources.’ (lines 379-381)
- Added a ‘,’ in ‘Our findings agree with those of Chivenge et al. \cite{Chivenge2011}, who found […]’ (line 407)
- Removed a ‘,’ in ‘[…], who found that ORMR treatments with low-quality organic resources tend to favor positive interactions as opposed to high-quality resources’ (line 412)
- Corrected for American English: ‘This implies that an increase of biomass due to an improved soil fertility could only have benefited SOC […]’ (line 468)
- Removed a ‘,’ in ‘that there were not enough long-term studies included in the analysis that could potentially confirm the arguments of Gentile et al. \cite{Gentile2011}.’ (line 488)

**3. The conclusions should be precise and to the point.**

We rephrased the conclusion in an attempt to be more precise and to the point (lines 552-583):

“Meta-analyzing 40 short- and long-term maize nutrient trials across SSA, revealed that compared to sole organic (OR) and mineral N (MR) applications the combined application (ORMR) does have a significant positive effect on maize grain yield productivity and AE of N, albeit more pronounced at higher N rates and for high-quality organic resources. For yield variability and SOC, however, the combined effect is negligible and treatment effects are mainly determined by the organic resource rate and quality. The N rate and organic resource quality were found to play an important role overall, such that compared to low-quality organic resources, increasing amounts of high-quality resources allow for (i) an increased productivity and AE, (ii) a less rapidly declining AE with N rate, (iii) a decreased yield variability, and (iv) an increased SOC.

While the ISFM combined application may not unequivocally outperform the sole applications on individual CSA pillars, the practice has been found to be appropriate for simultaneously and optimally addressing all three pillars and hence can be considered climate-smart. This paper provides evidence for the need of managing both mineral and organic resources in a holistic and integrated approach, such as aspired by the ISFM framework.”

**4. The use of abbreviations in not consistent throughout the manuscript. For eg. what is MR in line 66? What is ‘AND’ in lines 98 and 99?**

- MR indeed appeared twice in line 66, and should have been ‘mineral N’. This has been corrected (lines 69-70).
- The ‘AND’ and quotation marks surrounding multiple word search items were Google search syntaxes. These have been by commas and single quotes for all search items instead, respectively (lines 101-103).

The use of other abbreviations has been checked and improved accordingly:

- ‘dSOC1’ in Tables S2 and S3 in supporting information (both in table caption and content) were replaced by ‘dSOC’ which has correctly been introduced earlier in the manuscript.
- ‘dSOC’ has been properly introduced in the caption of fig 9 as “The output of the N-SOC model is used to predict changes in soil organic carbon from its initial values (dSOC) […]”.
- ‘dSOC’ has been properly introduced in the caption of fig 10 as “The output of the C-SOC model is used to predict changes in soil organic carbon from its initial values (dSOC) […]”.
- ‘YIELD’ has been properly introduced as “The linear mixed effect model for the yield analysis (YIELD)” (line 181).

**5. Expand ‘PRISMA’ in line 103.**

Done (lines 115-115).

**6. What exactly do the x and y axis of the graph in figure depict? What are the units and values? Where is statistical analysis for this graph?**

The figure number is not specified, but perhaps the reviewer is talking about Fig 2 or Fig 3.

- Fig 2 is a conceptual yield response curve, and therefore we argue units and values are not relevant. There is no statistical analysis as the graph merely intents to illustrate the hypothetical relative position of different response curves. In an attempt to make it clearer, the word ‘hypothetical’ has been replaced by ‘conceptual’ in the Fig 2 caption
- For Fig 3, we clarified the y axis label by changing it to ‘Scaled count data for each subtreatment’. We also changed the caption to ‘Distribution of data points, represented by scaled count data for each subtreatment across total N rate, applied as mineral or organic fertilizer’, and edited the first sentence of the paragraph to ‘The frequency distribution of yield data across total N rates gives an indication about the amount of data available by N rate, as shown by the density graph of Fig \ref{fig:Fig_3}.’ (line 278).

**7. The presentation of data in form of tables or figures is poor. There are too many figures and tables for the given discussion. Some of the figures can be combined.**

In an attempt to improve the presentation of data in form of tables or figures, we:

- Improved consistency by capitalizing the legend titles of Fig 3, 6, 7 and 8 from ‘treatment’ to ‘Treatment’
- Merged the legend for treatment colors and shapes of Fig 4
- Rephrased the reference to Table 1 as such: ‘An overview of the relevant data from the publications selected for each analysis is given in Table \ref{tab:Table_1}’ (lines 119-120)
- Rephrased the caption of Table 1 as such: ‘Overview of the data set used for the YIELD and SOC meta-analyses, with columns for the study*country*site combination index (id), study (reference), aridity index class (AI class)*, time span of the study’s experiment (time span), number of yield data (nYield), number of SOC data associated with C and N rates (nSOC C input and nSOC N input, respectively)\\(*) The site’s aridity index was extracted from the CGIAR-CSI Global-Aridity and Global-PET Database \cite{Trabucco2009}’
- Rephrased the caption of Table 2 as such: ‘List of organic resource types and associated quality classes, with columns for the respective number of studies (nReferences), number of yield data (nYield), and number of SOC data with C and N rates different from zero (nSOC C input and N input, respectively)’
- Rephrased the paragraph referring to Fig 4 as such: ‘Fig \ref{fig:Fig_4} shows the average yields of the different treatments, across all study sites and for each individually. The graph reveals that on average ORMR treatments tend to represent higher yield averages, followed by MR and OR, respectively. The control treatments represent, as expected, the lowest averages.’ (lines 283-284)
- Rephrased the first sentence of the Fig 4 caption as such: ‘(left) Overview of reported maize grain yield (t ha\textsuperscript{-1}) data, averaged by publication, site, and treatment, and ranked following increasing ORMR yields.’
- Rephrased the first two sentences referring to Fig 5 as such: ‘With the reported yield data, one can calculate the AE across total N input and by treatment and organic class (Fig \ref{fig:Fig_5}). From these calculations it is observed that the AE decreases in value as well as in variability with increasing N input.’ (lines 287-289)
- Added the AE abbreviation for agronomic efficiency to the caption of Fig 5 as such: ‘The agronomic efficiency (AE, in kg kgN\textsuperscript{-1}) across a total N input range of 0-200 (kg ha\textsuperscript{-1}), applied as mineral or organic fertilizer, and segregated by treatment and organic input quality class}’
- Explained the RMSE abbreviation in the last sentence of the Fig 6 caption as such: ‘The root mean square error (RMSE, in t ha\textsuperscript{-1}) is given for the different predicted yields from treatments with the respective organic classes’
- Added the AE abbreviation for agronomic efficiency to the caption of Fig 7 as such: ‘The agronomic efficiency (AE, in kg kgN\textsuperscript{-1}) was plotted for the available total N rate, within a range of 0-200 (kg ha\textsuperscript{-1}), applied as mineral or organic fertilizer, separately for each organic class and for ORMR with three different organic N input proportions’
- Explained the SD abbreviation in the Fig 8 caption: ‘Yield standard deviation (SD) responses across time are shown for each subtreatment, and relative to their respective predicted yield values.’
- Added space between x-axis title and graph of Fig 8
- Explained the RMSE abbreviation in the last sentence of the Fig 9 caption as such: ‘The root mean square error (RMSE, in \%) is given for the different predicted dSOCs from treatments with the respective organic classes’
- Explained the RMSE abbreviation in the last sentence of the Fig 10 caption as such: ‘The root mean square error (RMSE, in \%) is given for the dSOC predictions of the different subtreatments’

We politely disagree with “There are too many figures and tables for the given discussion. Some of the figures can be combined”. Our arguments are as follows:

- Both tables 1 and 2 briefly give a necessary overview of the data used for the analysis.
- All figures illustrate an essential element of the analyses:
  - (Fig 1 is required by the journal)
  - Fig 2 we believe is necessary to illustrate and visualize the rather complex term ‘interactive effects’ in order for to make it easier for the reader to understand.
  - Fig 3 explains why organic quality class IV could not be included in the YIELD analysis, and illustrates the importance of including N rate as a covariate in the different meta-analyses.
  - Fig 4 presents an overview of the raw, non-modelled data, and indicates the need for modelling
  - Fig 5 illustrates the importance of including organic quality class and N rate into the mixed effect modelling
  - Figures 6-10 each show a specific result of the different analyses, as brief and informative as possible.
- Further combining of Figures (such as was done for Fig 4), we believe would greatly diminish the clarity of the graphs, and overload the reader with distinct pieces of information.

**8. What is the difference between class I and II, and class III and IV manures? Explain clearly the difference in the composition.**

We integrated an explanation of the classification and different compositions as such: “These were categorized into four quality classes according to the organic data base (ODB) of Palm et al. (2001). High-quality organic classes I and II have a high N content (>2.5%), and low-quality classes III and IV have a low N content (<2.5%). Classes I and III have low lignin contents (<15%), whereas classes II and IV have high lignin contents (>15%) (Table 2).” (lines 146-149).

Furthermore, in the abstract I replaced “the organic resources of class I” by “the highest quality organic resources”

**9. What do you mean by dSOC?**

Is now being introduced and better explained in the following sentence: “The SOC data of a publication were considered relevant when at least two measurements over time were reported, so that responses could be calculated by subtracting the initial SOC from the SOC at a given time during or after the experiment (dSOC).” (lines 164-165). Therefore, the original introduction of ‘dSOC’ was removed and replaced by “Two separate linear mixed effect models were designed to model dSOC, […]” at lines 236-237.

**10. Based on the results, what kind of treatment would the authors suggest for better yield and productivity in SSA? Please explain in discussion.**

The following paragraph was added to the discussion (lines 490-514):

“We have seen that compared to the sole applications, the combination application has a significant positive effect on maize yield productivity and AE of applied N, which is more pronounced at higher N rates. For yield variability, but especially for SOC, the difference of the combined with the sole organic application was marginal. One can therefore not state that the ISFM combined application decreased yield variability or increased SOC compared to the sole organic and mineral N applications. It has been shown, however, that while the ISFM combined application may not outperform the sole applications on individual CSA pillars, the practice is necessary to simultaneously and optimally address all three pillars. First, mineral N proved to be necessary to achieve the highest productivity and AE for N rates < 100 kg N ha-1. This is important since the global average in 2013 was only 74 kg N ha-1, but it is particularly relevant in the context of smallholder farming in SSA, where mineral N use is even lower yet most yield production increase is needed (LuTian2017, Pasley2020). Within this N input range, the AE of total applied N would decrease when mineral N is combined with organic inputs, but this is limited when high-quality resources are used. Second, when considering the other two aspects of CSA, we observe that high quality organic inputs become necessary to achieve the lowest yield variability and greatest SOC increase, independent if in combination with mineral N. In summary, the findings of this paper therefore suggest that mineral N is necessary for the best low-input productivity, while high quality organics are necessary for achieving the lowest variability (class I or II) and highest SOC response (class I, II, or Manure). Combining the mineral and organic resources is therefore a way to achieve a win-win-win situation, where productivity, variability, and SOC are all three improved and optimized. For high-input scenarios, the case of combined application is even stronger, as it will outperform the mineral N fertilizer application effects on productivity.”

Based on the above points, the manuscript may be accepted for publication after ‘Major Revision’.

Reviewer #2: The paper tackles an important issue i.e. Integrated Soil fertility Management in sub-Saharan Africa through combining organic and mineral fertilisers. The authors carried a meta-analysis of short and long-term maize nutrient trials in sub-Saharan Africa so as to evaluate the effect of combined versus sole application of organic and mineral N inputs on (i) maize productivity and Agronomic Efficiency of Nitrogen, (ii) maize yield variability, and (iii) on SOC.
Overall, the manuscript is clearly, concisely and well written. The introduction is relevant and does provide sufficient justification for the review. Moreover, the materials and methods section is also well written. Consistent with the rest of manuscript, the results were also clearly explained and discussed. The figures used, although some not too clear, are relevant and assist in understanding the results better. The conclusions adequately address the objectives set out in the introduction.
Minor Corrections:

**Line 13: The sentence does not read well, consider deleting the conjunctive adverb "however".**

I deleted ‘however’, which makes the sentence indeed more readable (line 13).

**Line 20: it is not particularly clear what "its' refers to in this sentence**

I rephrased the sentence into two sentences and without ‘its’: ‘Yet, maize productivity in SSA is low and is being exacerbated by climate change \cite{Cairns2013, Tesfaye2015, Lipper2014}. Maize grain yields on average are 58% below the water-limited potential across tropical Africa \cite{Hillocks2014}, and are projected to decline by up to 12% by 2050 \cite{JonesThornton2003, Tesfaye2015}. ’ (lines 20-24)

**Line 23: Add "by' before up to.**

Done, see previous comment (line 24).

**Line 268: Replace "about" with of.**

Done (line 278).

**Line 290: Replace 'is depending" with "depends"**

Done (line 302).

**Line 296: Replace 'is depending" with "depends"**

Done (line 308).

**Line 377: Add "of" after the word 'importance"**

Done (line 389).

We trust these corrections and responses to the reviewers’ comments are satisfactory, and we appreciate your consideration of the revised manuscript. We are, however, available for further clarification or revisions if necessary.

Kind regards,

Gil Gram, on behalf of the authors of the manuscript
